# International variation in prescribing antihypertensive drugs: Its extent and possible explanations

**DOI:** 10.1186/1472-6963-5-21

**Published:** 2005-03-11

**Authors:** Atle Fretheim, Andrew D Oxman

**Affiliations:** 1 Informed Choice Research Department, Norwegian Health Services Research Centre, P.O. Box 7004, St. Olavs plass, Oslo, Norway

## Abstract

**Background:**

Inexpensive antihypertensive drugs are at least as effective and safe as more expensive drugs. Overuse of newer, more expensive antihypertensive drugs is a poor use of resources. The potential savings are substantial, but vary across countries, in large part due to differences in prescribing patterns. We wanted to describe prescribing patterns of antihypertensive drugs in ten countries and explore possible reasons for inter-country variation.

**Methods:**

National prescribing profiles were determined based on information on sales and indications for prescribing. We sent a questionnaire to academics and drug regulatory agencies in Canada, France, Germany, UK, US and the Nordic countries, asking about explanations for differences in prescribing patterns in their country compared with the other countries. We also conducted telephone interviews with medical directors of drug companies in the UK and Norway, the countries with the largest differences in prescribing patterns.

**Results:**

There is considerable variation in prescribing patterns. In the UK thiazides account for 25% of consumption, while the corresponding figure for Norway is 6%. In Norway alpha-blocking agents account for 8% of consumption, which is more than twice the percentage found in any of the other countries. Suggested factors to explain inter-country variation included reimbursement policies, traditions, opinion leaders with conflicts of interests, domestic pharmaceutical production, and clinical practice guidelines. The medical directors also suggested hypotheses that: Norwegian physicians are early adopters of new interventions while the British are more conservative; there are many clinical trials conducted in Norway involving many general practitioners; there is higher cost-awareness among physicians in the UK, in part due to fund holding; and there are publicly funded pharmaceutical advisors in the UK.

**Conclusion:**

Two compelling explanations the variation in prescribing that warrant further investigation are the promotion of less-expensive drugs by pharmaceutical advisors in UK and the promotion of more expensive drugs through "seeding trials" in Norway.

## Background

In many countries there is a substantial potential for savings if less expensive drugs, particularly thiazides, are prescribed rather than the more expensive drugs for hypertension [1]. The potential savings in the UK are £132 million ($200 million) per year (£2.22 ($3.36) per inhabitant; figures from the year 2000). The US and Norway could potentially save even more per inhabitant (£3.21 ($4.86) and £3.55 ($5.38) respectively, year 2000). An important reason for these differences in potential savings is that thiazides are used more in the UK than in the US and Norway. In this article we describe and attempt to explain international variation in prescribing patterns of antihypertensive drugs.

## Methods

We had access to sales figures for anihypertensive drugs for six countries (Canada, France, Germany, Norway, the UK and the US) for the year 2000. We also had survey-based information describing the diagnoses for which the drugs were being prescribed. This was relevant since antihypertensive drugs are also used for other indications, such as heart failure (e.g. ACE-inhibitors) and post-myocardial infarction (e.g. beta-blocking agents). The information was provided by IMS-Health.

The sales-figures were originally expressed as physical units (kg), which we transformed to defined daily dosages/1000 inhabitants/day [2]. The defined daily dose (DDD) is the assumed average dose used for a drug [3].

For each drug-class we estimated the total consumption by summarizing the consumption for each drug within a class. The total consumption for each class was then multiplied with the proportion of prescribing that was done specifically for hypertension. We estimated the consumption of the various drug classes for each country, and compared them.

The following drug-classes (and ATC-numbers) were included: alpha blocking agents (C02C A), thiazides (C03A, C03B og C03E), beta blocking agents (C07), calcium channel blockers (C08), ACE-inhibitors (C09A), ACE-inhibitors combined with a diuretic (C09B), angiotensin II antagonists (C09C), and angiotensin II antagonists combined with a diuretic (C09D).

We also obtained official sales statistics for antihypertensive drugs in the Nordic countries (Denmark, Finland, Iceland, Norway and Sweden) for 1999, and compared the patterns of consumption [4]. For these countries we did not adjust for the proportion of prescribing being made specifically for hypertension, as we did not have access to such information.

**Figure 1:**
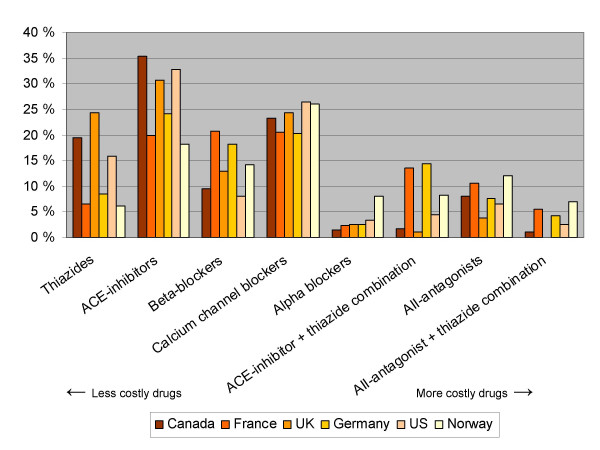
Consumption of drugs for the treatment of hypertension for six countries (percentage distribution within each country) *. *Consumption as defined daily dosages/1000 inhabitants/day. Based on figures on sales (year 2000) and indications for prescribing from IMS-Health

**Figure 2:**
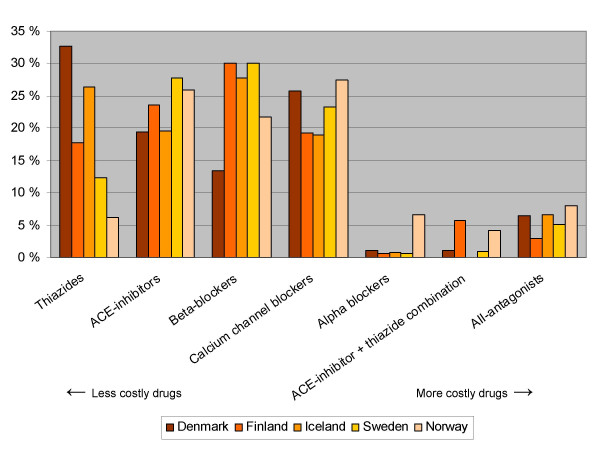
Consumption of antihypertensive drugs within the Nordic countries (percentage distribution within each country)* *Consumption as defined daily dosages/1000 inhabitants/day. Based on official sales statistics from 1999 [4]. No adjustment made for the relative proportion of prescribing being done for other indications than the treatment of hypertension

We circulated those results (figures 1 and 2) to a convenience sample of one academic in each of the included countries and asked about possible reasons for inter-country variation in prescribing patterns. The results were also sent to the drug regulatory agency in each country. The recipients were asked to answer the following five questions:

1. Are there specific policies, rules or regulations in place in your country that may influence the choice of drug in the treatment of hypertension (e.g. pricing policies, treatment-protocols etc.)?

2. What do you think are the main factors that influence the choice of drug in the treatment of hypertension in your country?

3. In particular, why do physicians prescribe newer, expensive drugs, such as calcium channel blockers or angiontensin II receptor blockers?

4. Similarly, why do physicians prescribe older, less expensive drugs, such as thiazides or beta-blockers?

5. We have attached a graph illustrating the profile of sales of antihypertensive drugs for some countries, including yours. How would you explain your country's profile in comparison with the other countries?

Lastly, we contacted the medical directors of the British and Norwegian affiliates of four major drug companies with antihypertensive drugs, and conducted a semi-structured telephone interview exploring possible explanations for the differences between these two countries, which had the largest differences in prescribing patterns (See additional file 1: Interview guide).

## Results

Calcium channel blockers and ACE-inhibitors are generally the most widely used drugs for the treatment of hypertension (figure 1 and Additional file 2: Sales of antihypertensive drugs, IMS). Apart from that, there is large variation among the countries with regards to the use of drugs for the treatment of hypertension.

In the UK thiazides account for 25% of consumption, while the corresponding figure for Norway is 6%. In Norway alpha-blocking agents account for 8% of consumption, which is more than twice the percentage found in any of the other countries. The use of combination drugs in the UK is strikingly low. The prescribing patterns also vary largely within the Nordic countries (figure 2 and Additional file 3: Sales of antihypertensive, Nordic countries).

The overall consumption of antihypertensive drugs is considerably higher in the US than in Canada, France, Norway or the UK (table 1).

**Table 1:** Consumption of antihypertensive drugs in six countries, based on sales figures provided by IMS-Health (year 2000)

	**Total consumption of antihypertensives (DDDs/1000 inhabitants/day)**	**Consumption of antihypertensives for the treatment of hypertension (DDDs/1000 inhabitants/day)**
Canada	163.6	119.6
France	171.7	133.4
Germany	205.9	145.3
Norway	171.6	115.7
UK	170.6	105.8
US	225.5	165.3

### Possible explanations for inter-country variation

We received answers from researchers in all the ten countries and drug regulatory agencies in Finland, Norway and Sweden and from a Department of Health official in the UK.

There was nearly full consensus among our respondents that marketing by pharmaceutical companies is the main explanatory variable for prescribing choices, and particularly the driving force behind the prescribing of newer drugs, such as calcium channel blockers and angiotensin II receptor blockers.

There was some variation among the respondents regarding the policies, rules and regulations related to prescribing of antihypertensive drugs in the various countries, but few believed that this had a major impact on prescribing. The exceptions were the Finnish respondents, who believed that their national clinical guidelines program could have had an impact, and the UK Department of Health official who considered central guidance ("especially NICE guidance") a major factor.

The answers given to our question on why doctors prescribe older drugs was mixed. Some researchers pointed to price considerations (Denmark, Finland, Canada, US), and some made statements like "Some physicians prescribe according to evidence-based principles, but they are probably a minority" (Canada, France). There were several possible explanations for the differences in prescribing patterns between their own and the other countries:

...."it is MOST unusual for UK GPs to use combination preparations. It is regarded as poor practice." (British researcher)

"The very low usage of thiazides probably reflects the views of the prominent specialists and researchers in this field in Norway" ...... "Many of the experts are deeply involved in industry sponsored research and tend to favour new products and their own research." (Norwegian drug regulator)

"Denmark's relatively large share of thiazides may to some extent be explained by factors like domestic production"...."but may also reflect their better system for continuing drug education among their GPs (e.g. academic detailing). In Norway this education is almost entirely left to the pharmaceutical companies." (Norwegian researcher)

"Beta-blockers have a long tradition in Sweden." (Swedish researcher)

"I once asked an older cardiologist about the reason for this difference and the answer was that there was no major tradition for beta-blockers in Denmark" (Danish researcher)

"The most striking difference for Canada is the lack of combination use of thiazides with ACEI and ARBs. This is probably due to the fact that these combination drugs are not funded in many of the provinces. This leads to a seemingly greater use of thiazides and ACEIs alone." (Canadian researcher)

Seven of the eight drug company medical directors (including two alternates) agreed to be interviewed: four from Norway and three from the UK.

Differences in physicians' attitudes were thought by all the directors to partly explain the differences in prescribing between the UK and Norway; e.g. that British doctors generally are conservative and slow implementers of new interventions while Norwegian physicians are "early adopters":

"My perception is that we Norwegians are, in general, "early adopters" – we are attracted to the new and "hot", be it kitchen equipment or other things." (N2)

"UK is in general a conservative country when it comes to prescribing new drugs. New therapies are slowly taken up" (UK1)

"I think this [low use of combination drugs] reflects the general conservatism that is typical of the British." (N2)

"Could be that the British doctors are more conservative – it wouldn't be surprising knowing how conservative the British are, in general!" (N4)

"It is, perhaps, true that British doctors comply more with guidelines than their Norwegian colleagues. The British are perhaps more respectful towards authorities while the Norwegians are more individualistic in their attitudes." (N4)

"It may be true that Norwegian tend to disregard clinical guidelines and rather adhere to their own convictions. ......It is typically Norwegian that each individual has his own personal opinion." (N2)

"There is usually a slow uptake of newer drugs in the UK. New data typically doesn't impact immediately – unless they're very strong." (UK2)

"One new trial result is not enough for a UK-doctor – again a consequence of the conservatism we have." (UK3)

The Norwegian directors also believed that conducting trials in general practice has an impact on prescribing and that a substantially higher number of physicians involved in such trials in Norway could explain differences in prescribing patterns:

"Many trials are being run in Norway."..... "I believe that the hand-on experience doctors get from participating in the trials increases their awareness and influences practice." (N1)

"I do believe that running a study like this is, by itself, of value from the marketing perspective." (N2)

"Of course we conduct clinical trials with the aim of developing newer and better drugs, but a marketing effect is unavoidable, and is an added benefit for the company." (N2)

"Yes, involvement by doctors in the running of clinical trials probably has an impact. In Norway a much higher proportion of GPs participate in trials compared to, for instance, the UK." (N2)

"I don't think that is true [that the differences can be explained by the higher number of trials that are conducted in Norway]. I can only speak for our company, but we have run several trials in UK general practice – often in collaboration with sites in Norway and the other Scandinavian countries. So this does not fit with my understanding." (UK1)

"Norwegian physicians are in general rapid implementers of new therapies. A major explanation for this is probably the high number of large clinical trials that are run in Norway. Relative to our population the number of patients participating in clinical trials is about the double of what can be expected compared to other countries." (N3)

"Norway is a key area for clinical drug trials, e.g. studies of organoprotective effects of antihypertensive drugs. This has been driving prescribing to the right in the graph [i.e. towards the more expensive alternatives]. Recently we have seen a slight increase in the use of ARBs, for the treatment of migraine. Again, an example of high quality trial results being rapidly taken up in clinical practice." (N3)

"I don't think participating in trials will lead to a change in prescribing habit for a UK-doctor." ...... "We do [run clinical trials where GPs participate] ... but maybe we do it less than in Norway." (UK3)

British doctors were also believed to be more cost-aware, mainly due to local budgets which include drug expenditures, thus driving prescribing towards less costly alternatives:

"In the UK they have given budget-responsibility to the local GPs, which could limit spending on drugs, for instance." (N1)

"It's probably true that UK physicians are more cost-aware. One reason may be their tradition of local budgets. Actually, I think Norwegian doctors have become less cost-aware now than they were before. At least that's the impression I have from speaking to our representatives – they tell me that the doctors put very little emphasis on price." (N2)

"Cost considerations are also part of the picture. It is my impression that UK doctors are more cost sensitive. This has to do with the limited drug budgets for each practice, but I think UK doctors also are happy to prescribe thiazides and beta-blockers." (UK1)

"Maybe the British physicians are more cost-aware. I think Norwegian doctors have regarded the drug-reimbursement system as a bottomless pit. They may be concerned about the costs for their patients, but not the societal costs." (N4)

"I have mentioned drug formularies... Also, primary care trusts have budgets they are responsible for, which includes spending on drugs." (UK2)

"I don't think that UK GPs are particularly acquisition-cost aware, but the drugs that are included in their formularies have gone through a process of appraisal where cost-effectiveness is an important element." (UK2)

"It could be that in Norway you focus more on the patient in front of you. The UK-system is very cost-conscious." (UK3)

Local influence from pharmaceutical advisors was also mentioned as an important force that "pushes the use of thiazides – for pure cost reasons" (UK3). This "local guidance" was also seen as a reason why "UK-doctors are hard work for industry to influence" (UK3).

Views on the role of evidence among Norwegian and British physicians were contradictory. On the one hand the directors seemed to agree that there is a "bias for evidence-based medicine" (UK1) in the UK, but on the other hand the Norwegian directors claimed that physicians in Norway are very much focused on trial results and particularly hard endpoints, e.g. effects on mortality rates:

"Our impression is that the doctors in Norway put great emphasis on documentation – they look at the evidence. They want trials with hard end-points." (N1)

"I think there is a particular focus on large studies with survival endpoints among Norwegian doctors." (N2)

"I think Norwegian physicians are particularly interested in research and the results of clinical trials." (N2)

"I do have the impression that Norwegian physicians are focused on hard endpoints, but if there is any difference compared to British doctors I don't know." (N4)

"I think guidelines are important in explaining the UK prescribing pattern and there is a strong bias for evidence-based medicine. Thiazides and beta-blockers have been recommended as first line, and doctors have followed this recommendation." (UK1)

"UK doctors may be more difficult to persuade, perhaps, with their strong focus on evidence. They demand trials that have shown the effectiveness of drugs on hard end points – their clinical usefulness." (UK1)

"Also in the UK some physicians want hard end-points – especially the physicians in secondary care. Many GPs are more focused on controlling the blood pressure." (UK2)

"Maybe Norway is less evidence-based and more patient-focused? ..... There is a fundamental belief in endpoints and outcomes – there is a hang-up on this in the UK." (UK3)

The British directors agreed that the low use of combination drugs in the UK was due to therapeutic traditions largely seeded at medical school. One of them also believed that "the general push for generic prescribing plays a role, since the combinations usually are branded drugs" (UK3).

The high use of alpha-blockers in Norway was considered to be an artefact by some since current regulation only allows for the reimbursement of such drugs if they are used for hypertension, and not for benign prostate enlargement. However, one of the directors refuted this, citing a study conducted among general practitioners where only 5–10% of reimbursed prescriptions for alpha-blockers were made for individuals without hypertension.

Two Norwegian directors confirmed that the relationship between specialists and industry might be a factor:

"Conflicts of interest of opinion leaders may be an issue" ...... "Norway has a rather high number of opinion leaders in the cardiovascular area that have been active in collaboration with industry." (N2)

"Their engagement with industry is important for the services they provide to their patients, e.g. the chance of accessing new therapies, more intense follow-up of patients etc. This is less so in the UK. For this reason industry may find it more difficult to convince physicians in the UK with their arguments." (N3)

"To get opinion leader endorsement is important. They rarely go against established guidelines, so they generally support the use of thiazides. Also the opinion leaders put great importance on cost-effectiveness, while tolerability for instance is less emphasised. Regarding their links to industry I think they are cautious – they have their integrity to maintain." (UK3)

"One difference might by that Norwegian opinion leaders seem to be somewhat less keen on promoting a specific drug – they worry about their credibility." (N2)

"In fact, my guess is that marketing in general – also the use of opinion leaders – is more aggressive in the UK, if there is any difference at all." (N1)

One Norwegian director mentioned the role of patients: "In the UK I think the doctor-patient relationship is more traditional, with the physician deciding for the patient, while here the patients are more involved in decision-making; and when given the choice they prefer the 'latest model"' (N1).

Financial incentives were mentioned by one UK director: "In the UK there are local prescribing incentive schemes that reward generic prescribing, reward high use of drugs in accordance with guidelines, etc. The reward can be money, which goes to the clinic. The GPs are independent contractors – running their own business." (UK3)

One director suggested that a higher threshold for initiating treatment among Norwegian physicians means that a higher proportion of those who are treated need more than one drug, and that this influences the overall prescribing pattern.

## Discussion

We have based our analysis on a cross-sectional data from one year. This may represent an incomplete picture of the prescribing in the ten countries since we are not able to detect prescribing trends, which may vary across countries. Another potential weakness of our analysis is that we have not taken epidemiological differences into account. For instance, a lower use of ACE-inhibitors in the US may be appropriate given the high number of Afro-Americans and the fact that these drugs are less effective in persons of African origin. Interestingly, however, the use of ACE-inhibitors in the US is particularly high.

There is considerable variation in prescribing patterns of antihypertensive drugs among the countries included in this study. The UK and Norway are at opposite ends of the spectrum.

The high use of alpha-blockers in countries such as Norway and the US is of concern since evidence in support of these drugs is particularly weak. The first major comparative trial of alpha blocking agents and other antihypertensives was stopped early due to the high rate of cardiovascular disease events among those allocated to the alpha-blocker [5]. Despite this, the sales of alpha-blockers in Norway have only gone down slightly (from 8.8 DDDs/1000 inhabitants/day in 2000 to 7.8 DDDs/1000 inhabitants/day in 2003) [3]. In the US the publication of the trial results appears to have had a greater impact on physicians' prescribing habits [6].

Not only do Norwegian physicians tend to choose drugs from the most expensive drug-classes, they also tend to select the more expensive drugs within a class (table 2). For example, metoprolol is the most sold beta-blocking agent in Norway and it is one of the most expensive drugs within its class [7]. Moreover, metoprolol is available from several manufacturers, and the most expensive version (Selo-Zok^®^) is the most sold [7]. This explains why beta-blocking agents have a higher average price than ACE-inhibitors in Norway (table 2), despite the fact that most beta-blocking agents are much less expensive than ACE-inhibitors [7].

**Table 2:** Drug-costs for one year's treatment, in Norway (ranked according to price)*

	Drug cost*
Thiazide	$33 (£22)
ACE-inhibitor	$150 (£99)
Beta-blocking agent	$174 (£115)
Calcium channel blocker	$211 (£139)
Alpha blocking agent	$269 (£178)
ACE-inhibitor with thiazide	$270 (£178)
Angiotensin II antagonist	$288 (£190)
Angiotensin II antagonist with thiazide	$328 (£216)

*Based on prices to consumer. The costs were calculated by dividing total sales of each drug class by consumption (year 2000). It is assumed that the average dose used equals the defined daily dose (DDD)

If Norwegian physicians were to prescribe antihypertensive drugs similarly to Danish physicians, the annual expenditure would be reduced by £26 million ($US 40 million), which is about 30% of current spending on these drugs in Norway. Interestingly, it was pointed out 20 years ago that the Norwegian drug expenditures for antihypertensives would be reduced by 36% if Norway were to adopt a Danish profile [8].

It is widely believed that marketing efforts from the pharmaceutical industry have a major impact on physicians' prescribing [9]. Our informants agreed with this, but the directors we interviewed did not indicate that marketing strategies differ much from country to country. According to them companies base their marketing on global strategies that are adapted locally. However, it is possible that Norwegian physicians interact more frequently with drug company representatives, for instance, and that this may explain some of the variation in prescribing behaviour. We have collected data on the scope of outreach visits by industry representatives to general practitioners in Norway, but we have not found similar data for any of the other countries in this study. Thus, no comparison is possible.

Non-industry pharmaceutical advisors play an active role in trying to influence prescribing behaviour among UK primary care physicians. They use strategies such as locally developed guidelines, feedback on prescribing, and outreach visits. A similar system does not exist in Norway and this may be one important explanatory factor for the observed differences between the UK and Norway.

Our survey of researchers and drug regulators yielded few plausible explanations for the variation in prescribing patterns. It is unlikely that practice guidelines alone have had an important impact [10]. The higher use of thiazides in Denmark and the higher use of beta-blockers in Sweden may be related to domestic production of these agents in the respective countries, and the lack of reimbursement may help to explain the lower use of combination drugs in Canada. These explanations are not likely to explain other differences.

The medical directors from drug companies in Norway and the UK suggested that fund holding for general practitioners was an important reason for the higher use of thiazides in the UK. However, the findings from numerous evaluations addressing the effect of fund holding on prescribing do not necessarily support this belief [11].

The assertion that Norwegian physicians are less conscious about the societal costs of their prescribing compared to their British colleagues is difficult to validate. Jacoby and colleagues observed in their interviews with 56 general practitioners in the UK that there was considerable variation concerning the level of cost-consciousness [12].

There seemed to be a consensus among the Norwegian medical directors that physicians in their country are particularly focused on research findings, especially trials that measure effects in terms of hard endpoints. However, this does not fit with our impressions. For example, in recent debates over first line antihypertensive drugs metabolic effects (soft endpoints), not hard end points, have been at the centre of the debate. Medical directors from the UK also did not share the impression that British physicians show less interest in research findings or put less emphasis on hard endpoints than their Norwegian colleagues.

Statins are another group of drugs for which the national prescribing patterns vary greatly [13]. In their recent paper, Walley and colleagues speculated that a major reason why Norway has the highest use of statins in the region is "the involvement of Norwegian doctors in seminal trials" [13]. However, this does not explain the variation in consumption of antihypertensive drugs, since there are essentially no trials that provide evidence against the use of thiazides or beta-blockers. A better explanation may be that participation in trials as such, and not necessarily seminal trials, influences prescribing. Pharmaceutical companies may include marketing considerations when planning clinical trials, in particular those conducted after drug approval has been granted. Studies conducted after marketing-approval has been granted are sometimes labelled "seeding trials", as they may be designed to "seed" the use of a drug among physicians [14-16]. All the Norwegian directors in our study seemed to agree that trial-participation influences prescribing. Despite searches in several databases, we did not find research data to support or refute this belief. This is surprising considering concerns that have been raised over the impact of seed trials [15,16]. Norwegian doctors are typically paid a bulk sum in the order of NOK 5000 – 15000 (£425 – £1275; $800 – $2400) for every patient they enrol in clinical trials. The hourly fee for doctors participating as investigators in clinical trials in Britain is set at £193.50 ($360), according to an agreement made by the British Medical Association and the Association of the British Pharmaceutical Industry. This fee includes all relevant overheads (Richard Tiner, personal communication).

For trial-participation to be an effective marketing strategy it is necessary to recruit as many physicians as possible. Also, a double blind trial is not the best design if the idea is for the physicians to obtain "provider-experience" with a specific drug [14]. In fact, many of the recent trials of antihypertensive drugs have recruited a very large number of physicians, and a non-blinded design is common. National registers of ongoing clinical trials, which could provide data regarding the extent of seed trials and phase III randomised trials, are generally either non-existent or not accessible.

We are not aware of data that document that physicians in Norway have a higher threshold for initiating treatment than in the UK. The fact that the consumption of drugs for hypertension is somewhat higher in Norway suggests the opposite (table 1).

There was a common belief among the medical directors that UK physicians are more conservative than their Norwegian colleagues, and it was also suggested that this mirrors differences in attitudes that exist in general between the British and Norwegian societies. We have not identified research findings that support or challenge this hypothesis. However, Jacoby et al did observe that the British general practitioners they interviewed "consistently described themselves as cautious and conservative" [12].

Two additional hypotheses to explain inter-country variation were suggested during the review process of this paper: (1) the use of drug-samples and (2) educational interventions. There is evidence suggesting that both may be effective in influencing prescribing of drugs [17,18]. However, we do not have information about the relative use of such interventions in the countries we have studied.

## Conclusion

The fourfold contrasts in use of thiazide and alphablockers in Norway versus the UK suggests that relative risks of potentially inappropriate prescriptions to Norwegian hypertensive patients are in the vicinity of 4. Two compelling explanations for the observed variation that warrant further investigation are the promotion of less-expensive drugs by pharmaceutical advisors in UK and the promotion of more expensive drugs through seeding trials in Norway.

## Competing interests

Both authors are employed by the Norwegian government, which has substantial interest in containing the cost of health care.

## Authors' contributions

AF conceived the study, carried out the survey and interviews, prepared the first manuscript and contributed to all other aspects of the study. ADO contributed to the design of the study, to the interpretation of data, and made critical revisions to the manuscript.

## Pre-publication history

The pre-publication history for this paper can be accessed here:



## Supplementary Material

Additional File 1 List of questions used in semi-structured telephone interviews with medical directors, or their alternates, of pharmaceutical companies. Click here for file

Additional File 2 Sales figures for each drug-class, and proportions of drugs prescribed for hypertension, presented country wise (Canada. France, Germany, UK, USA, Norway). Based on IMS-data for the year 2000. Click here for file

Additional File 3 Sales figures for each drug class, presented country-wise (Denmark, Finland, Iceland, Sweden, Norway). Based on official sales statistics for the year 1999. Click here for file
