# Supplementary material for: International variation in prescribing antihypertensive drugs: Its extent and possible explanations
Source: BMC Health Serv Res. 2005 Mar 11;5:21. doi: 10.1186/1472-6963-5-21 (PMC1079831; doi:10.1186/1472-6963-5-21)
Supplement: Additional File 1 — List of questions used in semi-structured telephone interviews with medical directors, or their alternates, of pharmaceutical companies. [file 1472-6963-5-21-S1.doc]

# Interview guide, Medical Directors

Why do you think physicians in the UK have a prescribing pattern than their Norwegian colleagues? Particularly, why are the sales of thiazides so high in the UK compared to Norway?

1. Differences in marketing strategies?
2. What is your marketing strategy?
3. Are there differences in how difficult it is to influence physicians?
4. Are there differences in the degree of physician-involvement in clinical trials?
5. Do you conduct trials where GPs participate?
6. Are there differences in the views of national opinion leaders and in how the companies use this?
7. What are the views of national opinion leaders?
8. Are there differences in government policies?
9. Is the government more/less active towards physicians in influencing prescribing?
10. What is the government doing?
11. Are there differences in Continuous medical education programs?
12. Are there any – who runs them?
13. Are their differences regarding incentives for choosing various drugs?
14. Which incentives do you have?
15. Are there regulatory measures that can explain the variation?
16. Which regulatory measures do you have in your country?
