# Supplementary material for: International variation in prescribing antihypertensive drugs: Its extent and possible explanations
Source: BMC Health Serv Res. 2005 Mar 11;5:21. doi: 10.1186/1472-6963-5-21 (PMC1079831; doi:10.1186/1472-6963-5-21)
Supplement: Additional File 2 — Sales figures for each drug-class, and proportions of drugs prescribed for hypertension, presented country wise (Canada. France, Germany, UK, USA, Norway). Based on IMS-data for the year 2000. [file 1472-6963-5-21-S2.doc]

Sales of antihypertensive drugs (based on IMS-data for the year 2000)

|  | **Total use (DDDs*/1000 inhabitants/day)** | **Proportion prescribed for hypertension** | Sales for hypertension (DDDs*/1000 inhabitants/day) |
| --- | --- | --- | --- |
| **Canada** |  |  |  |
| *Thiazides* | 26.8 | 87 % | 23.3 |
| Alpha-blockers | 3.6 | 47 % | 1.7 |
| *Beta-blockers* | 21.9 | 52 % | 11.5 |
| *Calcium Channel Blockers* | 39.2 | 71 % | 27.9 |
| *ACE-inhibitors* | 58.0 | 73 % | 42.4 |
| *ACE-inhibitor + thiazide* | 2.3 | 91 % | 2.1 |
| *AII-antagonist* | 10.6 | 90 % | 9.6 |
| *AII-antagonist + thiazide* | 1.2 | 98 % | 1.2 |
| Total | 163.6 |  | 119.6 |
|  |  |  |  |
| **France** |  |  |  |
| *Thiazides* | 9.9 | 90 % | 8.8 |
| *Alpha-blockers* | 4.3 | 74 % | 3.1 |
| *Beta-blockers* | 41.7 | 67 % | 27.7 |
| *Calcium Channel Blockers* | 38.2 | 72 % | 27.5 |
| *ACE-inhibitors* | 35.5 | 75 % | 26.6 |
| *ACE-inhibitor + thiazide* | 19.3 | 94 % | 18.2 |
| *AII-antagonist* | 15.2 | 92 % | 14.0 |
| *AII-antagonist + thiazide* | 7.7 | 96 % | 7.3 |
| Total | 171.7 |  | 133.4 |
|  |  |  |  |
| **Germany** |  |  |  |
| *Thiazides* | 23.3 | 54 % | 12.6 |
| *Alpha-blockers* | 5.0 | 74 % | 3.7 |
| *Beta-blockers* | 40.6 | 66 % | 27.0 |
| *Calcium Channel Blockers* | 48.4 | 62 % | 30.1 |
| *ACE-inhibitors* | 47.8 | 75 % | 35.8 |
| *ACE-inhibitor + thiazide* | 25.4 | 83 % | 21.2 |
| *AII-antagonist* | 13.1 | 87 % | 11.4 |
| *AII-antagonist + thiazide* | 6.8 | 89 % | 6.0 |
| Total | 205.9 |  | 145.3 |
|  |  |  |  |
| **Norway** |  |  |  |
| *Thiazides* | 9.1 | 79 %**^†^** | 7.2**^†^** |
| *Alpha-blockers* | 10.5 | 89 %**^†^** | 9.4**^†^** |
| *Beta-blockers* | 32.6 | 50 %**^†^** | 16.3**^†^** |
| *Calcium Channel Blockers* | 50.4 | 60 %**^†^** | 30.2**^†^** |
| *ACE-inhibitors* | 35.0 | 60 %**^†^** | 21.0**^†^** |
| *ACE-inhibitor + thiazide* | 10.4 | 92 %**^†^** | 9.6**^†^** |
| *AII-antagonist* | 15.6 | 90 %**^†^** | 14.0**^†^** |
| *AII-antagonist + thiazide* | 8.1 | 100% **^†^** | 8.1**^†^** |
| Total | 171.6 |  | 115.7**^†^** |
|  |  |  |  |
| **UK** |  |  |  |
| *Thiazides* | 38.8 | 69 % | 26.9 |
| *Alpha-blockers* | 6.1 | 45 % | 2.8 |
| *Beta-blockers* | 28.7 | 47 % | 13.4 |
| *Calcium Channel Blockers* | 46.6 | 55 % | 25.4 |
| *ACE-inhibitors* | 42.6 | 75 % | 31.9 |
| *ACE-inhibitor + thiazide* | 1.3 | 85 % | 1.1 |
| *AII-antagonist* | 6.1 | 65 % | 4.0 |
| *AII-antagonist + thiazide* | 0.2 | 73 % | 0.1 |
| Total | 170.6 |  | 105.8 |
|  |  |  |  |
| **USA** |  |  |  |
| *Thiazides* | 32.5 | 81 % | 26.2 |
| *Alpha-blockers* | 11.6 | 48 % | 5.6 |
| *Beta-blockers* | 25.4 | 53 % | 13.4 |
| *Calcium Channel Blockers* | 57.5 | 76 % | 43.6 |
| *ACE-inhibitors* | 73.2 | 74 % | 54.1 |
| *ACE-inhibitor + thiazide* | 8.1 | 92 % | 7.4 |
| *AII-antagonist* | 12.7 | 85 % | 10.8 |
| *AII-antagonist + thiazide* | 4.6 | 93 % | 4.3 |
| Total | 225.5 |  | 165.3 |
|  |  |  |  |

*Defined daily dosages

†Proportions for Norway are based on data from 1994-96.
