# Supplementary material for: International variation in prescribing antihypertensive drugs: Its extent and possible explanations
Source: BMC Health Serv Res. 2005 Mar 11;5:21. doi: 10.1186/1472-6963-5-21 (PMC1079831; doi:10.1186/1472-6963-5-21)
Supplement: Additional File 3 — Sales figures for each drug class, presented country-wise (Denmark, Finland, Iceland, Sweden, Norway). Based on official sales statistics for the year 1999. [file 1472-6963-5-21-S3.doc]

Total sales of antihypertensive drugs in Nordic countries (based on official sales figures for the year 1999)*

|  | **Denmark** | Finland | Iceland | **Sweden** | **Norway** |
| --- | --- | --- | --- | --- | --- |
| *Thiazides* | 45.5 | 32.3 | 36.3 | 17.6 | 9.1 |
| *ACE-inhibitors* | 27.0 | 43.0 | 26.8 | 39.4 | 37.9 |
| *Beta-blockers* | 18.7 | 54.7 | 38.1 | 42.6 | 31.8 |
| *Calcium channel blockers* | 35.7 | 35.1 | 26.0 | 33.0 | 40.2 |
| *Alpha blockers* | 1.6 | 1.1 | 1.0 | 0.9 | 9.7 |
| *ACE-inhibitor + thiazide combination* | 1.5 | 10.4 | 0.0 | 1.3 | 6.0 |
| *AII-antagonists* | 8.9 | 5.3 | 9.2 | 7.2 | 11.8 |
| Total | 138.9 | 181.9 | 137.4 | 142.0 | 146.5 |

*Proportions prescribed for the treatment of hypertension are not available
